# Recovery and functional validation of hidden soil enzymes in metagenomic libraries

**DOI:** 10.1002/mbo3.572

**Published:** 2019-03-09

**Authors:** Dayana Calderon, Luis Peña, Angélica Suarez, Carolina Villamil, Adan Ramirez‐Rojas, Juan M. Anzola, Juan C. García‐Betancur, Martha L. Cepeda, Daniel Uribe, Patricia Del Portillo, Alvaro Mongui

**Affiliations:** ^1^ Molecular Biotechnology Research Group Corporación CorpoGen Bogotá Colombia; ^2^ Leibniz Institute for Natural Product Research and Infection Biology – Hans Knöll Institute Friedrich‐Schiller Universität Jena Germany; ^3^ Computational Biology Corporación CorpoGen Bogotá Colombia; ^4^ Institute for Molecular Infection Biology Universität Würzburg Würzburg Germany; ^5^ Biotechnology Institute Universidad Nacional de Colombia Bogotá Colombia; ^6^ Department of Biological Sciences Universidad de los Andes Bogotá Colombia

**Keywords:** Environmental microbiology, Functional genomics, Metagenomics, Microbial genomics

## Abstract

The vast microbial diversity on the planet represents an invaluable source for identifying novel activities with potential industrial and therapeutic application. In this regard, metagenomics has emerged as a group of strategies that have significantly facilitated the analysis of DNA from multiple environments and has expanded the limits of known microbial diversity. However, the functional characterization of enzymes, metabolites, and products encoded by diverse microbial genomes is limited by the inefficient heterologous expression of foreign genes. We have implemented a pipeline that combines NGS and Sanger sequencing as a way to identify fosmids within metagenomic libraries. This strategy facilitated the identification of putative proteins, subcloning of targeted genes and preliminary characterization of selected proteins. Overall, the *in silico* approach followed by the experimental validation allowed us to efficiently recover the activity of previously hidden enzymes derived from agricultural soil samples. Therefore, the methodology workflow described herein can be applied to recover activities encoded by environmental DNA from multiple sources.

## 1 INTRODUCTION

The total number of microbial cells on Earth's surface has been estimated at 4–6 × 10^30^ (Knight et al., 2012) and 3,000–11,000 distinct microbial genomes have been calculated per gram of soil (Sleator, Shortall, & Hill, 2008), making this environment one of the biggest reservoir of microbial diversity on the planet. The vast microbial diversity present in soils is an essential source of novel therapeutic agents (Singh & Macdonald, 2010) and compounds relevant for industrial applications (Beloqui et al., 2008). However, the fact that most of these microbes are nonculturable and therefore still uncharacterized, has hampered the development of large collections of novel bioproducts with direct application in biotechnology, agriculture, industry, and pharmaceutical processes. In the last decades, this panorama has changed, thanks to advances in our knowledge of the microbial world and the development of technological platforms aimed at the discovery and characterization of novel compounds from different sources including soils. Innovation in sequencing technologies together with novel software for bioinformatic analyses (Davenport & Tümmler, 2013; Scholz, Lo, & Chain, 2012), new tools for protein engineering, (Leisola & Turunen, 2007; Privett et al., 2012; Smanski et al., 2016), developments in high‐throughput screenings, and single‐cell analysis to cultivate previously nonculturable microbes (Ishii, Tago, & Senoo, 2010), among others, have opened new perspectives for finding new compounds and molecules in the microbial biodiversity.

In this sense, metagenomics, which involves direct analysis of DNA from environmental samples is a powerful methodology for the identification of novel compounds (Akondi & Lakshmi, 2013). In order to explore this potential, whole environmental DNA from both cultured and noncultured microorganisms is isolated and used to construct metagenomic libraries in well‐known bacterial species. These libraries are then subjected to function‐driven or sequence‐driven analyses. In the first approach, individual clones are screened using a suitable enzymatic substrate or assay. In the sequence‐driven approach, the metagenomic DNA is initially screened for particular DNA sequences using conserved primers or probes that are designed to identify the genes of interest. Both methodologies have been successfully used in metagenomic analyses to characterize potential industrial products (Hjort et al., 2014; Itoh et al., 2014; Verma & Satyanarayana, 2013). However, functional analyses are often problematic because the identification of genes and their subsequent activities depends on conditions that affect expression and detection, such as the selected host‐vector system, the size of the gene of interest, its abundance in the metagenomic source, the detection method used, and the efficiency of heterologous gene expression in the selected host (Ekkers et al., 2012). On the other hand, sequence‐driven approaches mostly rely on homologous sequences reported in databases (Ufarté, Potocki‐Veronese, & Laville, 2015) that are based on proteins already described, making the discovery of entirely new enzymes unlikely, especially for those where sequence can diverge significantly from already described families. Despite this, homology‐based analysis also allows the recovery of new enzymatic variants having extra advantages, like better ability to degrade a substrate or greater stability under adverse conditions (Lee & Lee, 2013; Simon & Daniel, 2011). In conclusion, the success rate of identifying novel compounds could be extremely low (Ekkers et al., 2012). The vast increase in data and tools now becoming available can gradually minimize this problem. For example, the coexpression of heterologous sigma factors in the host strain has improved the discovery of novel genes in a metagenomic library, therefore helping to overcome difficulties associated with heterologous expression (Gaida et al., 2015; Guazzaroni, Silva‐Rocha, & Ward, 2014; Rocha‐Martin et al., 2014).

In this study, we report a platform that combines next‐generation sequence (NGS) and bioinformatics tools to optimize the discovery of biotechnologically useful enzymes present in metagenomic libraries derived from soil. This strategy revealed a novel lipase/esterase and two proteases, enzymes that were not identified in traditional functional metagenomic screens. We suggest that the proposed pipeline can be applied to enhance efficacy of metagenomic library screens.

## 2 MATERIALS AND METHODS

### 2.1 Bacterial strains and growth conditions

*Escherichia coli* EPI300 strain (Epicentre, Madison, WI) was used as host for the construction of metagenomic libraries using pCC2FOS (Epicentre) as vector. For plasmid storage, *E. coli* OneShot TOP10 (Invitrogen, Carlsbad, CA) was used and recombinant protein expression was performed in *E. coli* BL21 DE3 and *E. coli* LMG‐194 strains (Invitrogen). Lysogenic Broth (LB) was used to grow all bacterial strains at 37°C in constant agitation, including either 12.5 μg/ml chloramphenicol for metagenomic library clones or 100 μg/ml ampicillin for plasmid maintenance and recombinant protein expression.

### 2.2 Soil sample collection

Rhizospheric soil samples were collected from three different *Solanum phureja* farms located in the Cundinamarca Andean Plateau, Colombia. Sampling sites were chosen for having similar conditions of climate and altitude (12°C–14°C and above 2,600 m above sea level). The specific farm names and sites locations were: Rosal (4° 50′ 60′' North; 74° 16′ 0′' West), Subachoque (4° 56′ 0′' North; 74° 10′ 60′' West), Tausa (5° 12′ 0′' North; 73° 52.60′ 60′' West) (Flórez‐zapata et al., 2013). The project was carried out in private lands and all the owners gave us permission to take the samples. Additionally, we confirm that sample collections did not involve endangered or protected species.

### 2.3 DNA isolation and metagenomic library construction

Metagenomic DNA extraction was performed with 8 g of a pooled sample from all collected soils using the UltraClean Mega Soil DNA Kit (MOBIO Laboratories, Carlsbad, CA), with some modifications to the manufacturer's protocol. Soil samples were subjected to 60°C–65°C to assure complete lysis of microorganisms and to obtain good quality DNA. Additionally, steps involving mixing by vortex were eliminated to prevent DNA fragmentation. The extracted DNA was concentrated in 5 mol/L sodium chloride–ethanol solution, and then eluted in Tris‐EDTA. DNA samples were separated by low‐point agarose gel electrophoresis at 30V during 16 hr. A 30‐kb fragment of high molecular weight (HMW) metagenomic DNA was selected and purified using QIAquick Gel Extraction Kit (QIAGEN GmbH, Germany) as previously reported (Prakash & Taylor, 2012). CopyControl Fosmid Library Production Kit (Epicentre, Madison, WI, USA) was used to construct the metagenomic library following manufacturer's instructions, using 0.25 μg HMW DNA and 0.5 μg of vector. The obtained metagenomic library (7,296 metagenomic clones) in *E. coli* EPI300 was stored at −80°C in 20% (vol/vol) glycerol‐LB media with chloramphenicol until used.

### 2.4 Sequencing strategy and contig assembly

Fosmid DNA from 40 randomly selected metagenomic clones was extracted using the FosmidMAX^™^ DNA Purification Kit (Epicentre). Once normalized, pooled samples were sequenced using 454‐FLX technology (Selah Genomics, University of South Carolina, USA). The resulting reads were cleaned from pCC2FOS vector and *E. coli* sequences (Genbank accession No. CP001637) by BLAST, using an *E‐*value threshold ≤ 1e^−5^ and coverage ≥ 70%. The final dataset was independently assembled using GS de novo Assembler software (v. 2.3, Roche Newbler, Branford, CT) and CLC Genomics Workbench (www.quiagenbioinformatics.com). Assembled contigs were submitted to Genbank under accession numbers MG564783 to MG565967.

### 2.5 Mapping of insert‐fosmid ends—TAGS

We performed Sanger sequencing to determine the ends of each insert for the 40 selected fosmids, allowing us to map the assembled contigs with their respective original bacterial clones. We refer to these FASTA insert‐ends as TAGS. Sequencing was performed using primers indicated in the CopyControl library production kit for pCC2FOS vector (FWD: 5′‐GTACAACGACACCTAGAC‐`3) and REV: 5′‐CAGGAAACAGCCTAGGAA‐`3), and the subsequent mapping of these TAGS to their respective contig was carried out using BLAST (Altschul, Gish, Miller, Myers, & Lipman, 1990).

### 2.6 ORF and gene‐protein feature predictions

Gene and Open Reading Frame (ORF) predictions of sequenced metagenomic inserts were carried out with MetaGeneMark (Zhu, Lomsadze, & Borodovsky, 2010) and EMBOSS suite (http://emboss.sourceforge.net ). Parameters for both programs were set to the prokaryotic genetic code, ATG, GTG, and TTG as start codons and TAA, TGA, and TAG as stop codons. Minimum gene length was set to 30 amino acids (aa). Gene predictions (putative proteins) were then searched against the PFAM database (http://pfam.xfam.org) using HMMER (Krogh et al., 1994) with cutoff *E‐*value of 1e^−10^, in order to determine their most likely functions as a result of the domains found in each case. Domains used to identify *in silico* lipases/esterases and proteases from the TAG‐assigned fosmids are included in Table S1. In case a putative gene was predicted for having both lipases/esterases and proteases domains, its activity was only evaluated based on the most significant *E*‐value score.

### 2.7 Gene ontology functional analysis

Predicted peptides and their respective PFAM domains were used to map to Gene Ontology (full GO) and GoSlim terms. AmiGO database (http://amigo.geneontology.org) was the source for Ontologies, particularly the ontology for metagenomics (goslim_metagenomics). Each PFAM domain present in our sample was mapped to full GO and then to GoSlim terms. Frequency analysis and chart were performed using GoSlim terms.

### 2.8 Subcloning and recombinant protein expression

A selected ORF (Consensus_gene_420) encoding the putative metagenomic lipase/esterase enzyme LipM, was amplified from its corresponding metagenomic clone (*E. coli* EPI300_ F5_C17) using Accuzyme (Bioline, London, UK) and the following primers: LipM‐F (5′‐CACCATGCCTGTCGATCAGCCA‐3′) and LipM‐R (5′‐CGCCGTTTTCCCGGAAGTGAC‐3′). PCR was carried out under the following conditions: 95°C for 5 min followed by 35 cycles of 95°C for 45 s, 65°C for 45 s, 72°C for 1 min and a final extension step of 10 min at 72°C. The PCR product was purified with the QIAquick PCR Purification Kit (Qiagen) and the purified fragment was cloned into pET100/D‐TOPO expression vector, following manufacturer's recommendations (Invitrogen). The putative metagenomic protease Prot1 coding gene (Consensus_gene_436) was amplified with the primers Prot1‐F (5′‐AActgcagGAACAATTCGAGCCCGAAG‐3′) and Prot1‐R (5′‐AActgcagTTGAGCAGATTCTCCCGAA‐3′) from clone *E. coli* EPI300_F8_C18. The putative metagenomic protease Prot2 coding gene (Consensus_gene_496) was amplified using the oligonucleotides Prot2‐F (5′‐AActgcagCGATGACCGATTCGACAA‐3′) and Prot2‐R (5′‐AActgcagTTCCAGTTTAGCGAACGC‐3′) from the bacterial clone *E. coli* EPI300_F38_C21. Recognition sites for *Pst*I restriction enzyme were included in these primers to facilitate the cloning process (lowercase on primer sequences). PCR condition for these protease‐encoding genes were as follows: 95°C for 5 min; 35 cycles of 95°C for 45 s, 60°C for 45 s, 2 min at 72°C; and a final extension step of 10 min at 72°C. Resulting PCR products were *Pst*I restricted and cloned into pBAD/gIII expression vector (Invitrogen). *E. coli* BL21 DE3 was used for the recombinant expression of LipM, while *E. coli* LMG‐194 (Invitrogen) was used for the recombinant expression of Prot1 and Prot2 proteins.

For recombinant protein expression, bacterial clones were grown in LB media supplemented with ampicillin until absorbance (OD_600 nm_) reached 0.5. Induction was carried out for five additional hours with 1 mmol/L isopropyl β‐D‐1‐thiogalactopyranoside (IPTG) or 0.2% L‐arabinose (Invitrogen). Bacterial cell lysis was performed with 0.1 mm diameter zirconia/silica beads in a Mini‐Beadbeater‐96 (Biospec Products, Bartlesville, OK), following a 3‐cycle protocol of 2‐min lysis and ice chilling for 10 min. Samples were centrifuged and the resulting supernatants (soluble fractions resuspended in phosphate‐buffered saline, PBS) and pellets (insoluble fractions resuspended in 6M urea) were evaluated by SDS‐PAGE and western blot using the anti‐polyhistidine monoclonal antibody against the 6xHis‐tag of the protein (Sigma‐Aldrich). Lipolytic or proteolytic activities of these subclones were assessed as described below, using the soluble bacterial extracts.

### 2.9 Purification of recombinant proteins

The recombinant proteins were purified from the whole bacterial extracts by affinity chromatography using a Ni^2+^‐ NTA resin (QIAGEN, CA, Hilden, Germany), according to the manufacturer's recommendations. Resin was equilibrated with PBS pH 7.0 with 15 mmol/L Imidazole. Nonretained fractions were eluted with the same buffer and, once the protein of interest was retained on the column, it was eluted in PBS with 250 mmol/L Imidazole. Resulting fractions were later analyzed by SDS‐PAGE and western blot.

### 2.10 Enzyme activity determination and characterization

Functional analyses of putative lipases/esterases and proteases present in the original metagenomic clones were performed by halo formation using conventional plate assays. For lipases/esterases, the screening was performed on LB‐Agar supplemented with 1% (wt/vol) tributyrin (Sigma‐Aldrich, Saint Louis, MO), while for the detection of proteolytic activity, a modified calcium caseinate agar media (Merck, Darmstadt, Germany) was used. Both activities were recorded after incubating the metagenomic clones in the selective media at 37°C for 2–3 days.

Metagenomic clones were grown until absorbance reached 0.8. Then bacterial cultures were centrifuged for 10 min at 6,000*g*. Bacterial pellets were resuspended in PBS buffer and lysis was performed using the Mini‐Beadbeater‐96, as described above. After lysis, samples were centrifuged and the resulting supernatants obtained.

For the enzyme activity determinations, both metagenomic clones and subclones extracts, as well as the purified recombinant proteins, were used. Lipolytic activity determination was performed by incubation of soluble bacterial extracts with the substrate *p*‐Nitrophenyl butyrate (0.5 mmol/L) (Sigma‐Aldrich) at 37°C for 20 min. Enzyme activity was quantified by absorbance at 410 nm, based on the release of *4*‐Nitrophenol using the TECAN GENios Spectrophotometer (Tecan, Männedorf, Switzerland). Extract of *E. coli* EPI300 was used as negative control.

Proteolytic activity was measured using casein as substrate and the colorimetric method of Folin Ciocalteu reagent (Sigma‐Aldrich). Briefly, 100 μl of soluble bacterial fraction was combined with 200 μl of 1% (wt/vol) casein and the resulting mix incubated for 1 hr at 45°C. The enzymatic reaction was stopped with 300 μl 5% (vol/vol) trichloroacetic acid and centrifuged for 10 min at 6,000*g*. Fifty microliter of sample supernatant was added to a mix of 100 μl of 500 mmol/L NaOH and 30 μl of 1:3 diluted Folin Ciocalteu reagent. The mix was further incubated at room temperature for 15 min and measured at 595 nm. A standard curve of tyrosine (0.110–1.5 μmol) was used to calculate the released tyrosine from the experimental samples. Extracts of *E. coli* LMG‐194 and *E. coli* BL21 DE3 were used as negative controls. One unit (U) of protease activity was defined as the enzyme quantity required to release 1 μmol of tyrosine per minute per mL. Results of proteolytic activity are shown in U/ml. Determination of optimal temperature, pH, and cofactors were also evaluated (Lee et al., 2007; Neveu, Regeard, & DuBow, 2011).

### 2.11 Nucleotide and amino acid sequences

Amino acid sequences of proteins Prot1, Prot2, and LipM, as well as their corresponding coding sequences (Clone 1, 2, and 3), can be found in NCBI database under the accession numbers MG272470, MG272471, and MG272472, respectively.

### 2.12 Statistical analyses

Nonparametrical Wilcoxon Test (one tailed) was used for the enzyme activity analyses of bacterial clones. A *p*‐value < .05 was considered to be statistically significant.

## 3 RESULTS AND DISCUSSION

**Figure 1:**
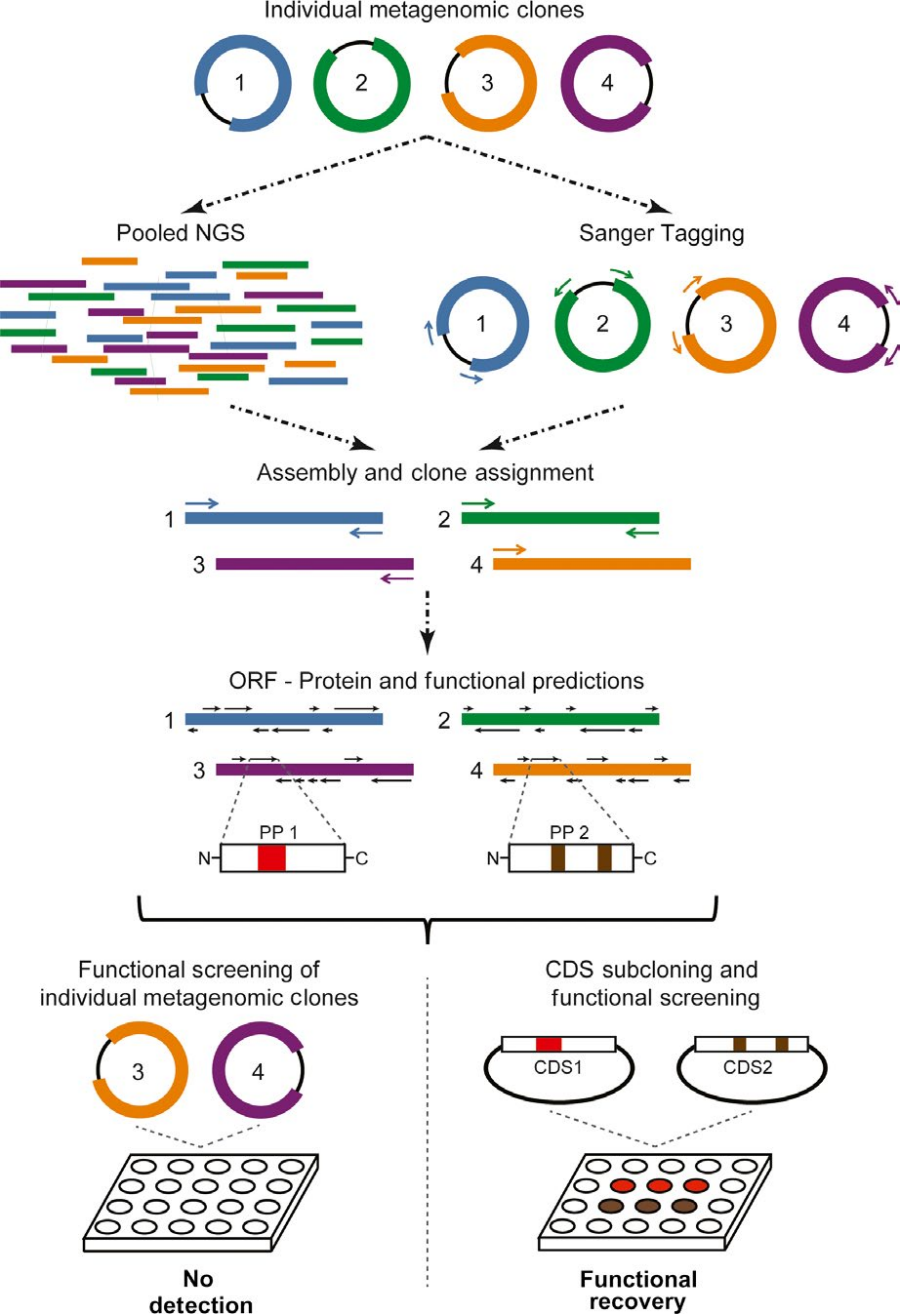
Pipeline overview. Isolated metagenomic clones are pooled in one sample for a massive sequencing analysis and independently analyzed by Sanger sequencing, in order to map the metagenomic inserts to their corresponding bacterial clones. After DNA assembly and clone assignment processes, ORF predictions and functional characterization of predicted putative proteins (e.g., PP1 and PP2) are performed. Selected coding sequences (e.g., CDS1 and CDS2) associated with the enzymatic activities of interest are matched to the original metagenomic clones or subcloned for independent maintenance in plasmid vectors. Finally, functional analyses on subclones expressing the predicted proteins allow the recovery of several enzymatic activities not identified in traditional functional metagenomic assays

Given the low probability of success in finding a gene of interest by functional metagenomic screens (Ekkers et al., 2012), we implemented a pipeline that incorporates sequence analyses to identify genes of interest. This study was performed on metagenomic DNA obtained from rhizospheric soils of the native potato *Solanum tuberosum* group *phureja*, a staple crop in Colombia (Rozo & Ramírez, 2011). The overall strategy is shown in Figure 1.

### 3.1 Metagenomic DNA sequencing and assembly

A small metagenomic library obtained from soil DNA from *S. phureja* crops consisted of 7,296 bacterial clones. Fosmids from 40 randomly selected metagenomic clones were sequenced using the 454‐sequencing technology (Roche), resulting in 135,103 reads with an average length of 369 nucleotides (nt).

After adapter trimming and vector‐host sequence removal, the remaining 85,745 sequences were assembled, obtaining a total of 37 contigs longer than 10 kb, which was the expected lower bound limit of our metagenomic library size (Table 1). Taking in consideration an average read size of 369 nt, the theoretical estimated coverage for each assembled fosmid was nearly 30X. The mapping of all the assembled contigs (> 1 kb) against the insert‐fosmid end sequences—TAGS of each of the 40 selected fosmids, resulted in 18 contigs successfully tagged to their corresponding fosmid in both ends (5′ and 3′ ends). In other words, these contigs included the full fosmid and the metagenomic insert sequences from 18 bacterial clones. Another 15 contigs were tagged to their corresponding fosmid in only one end, meaning that they were only partially sequenced (Table S2). Lam KN and coworkers (Lam et al., 2014) reported a similar approach with the difference that they used the Illumina HiSeq 2000 technology (90‐base paired sequencing), and were able to fully assemble 22 out of 92 (23.9%) metagenomic clones, while we fully assembled 18 out of 40 (45%). This higher percentage is most likely due to the longer sequence size obtained by the discontinued 454‐technology that favored our contig assembly process. The current Illumina MiSeq technology, which gives a read length up to 500‐600 nt, could be further included in this approach to obtain enough reliable DNA information from hundreds to thousands of pooled fosmid DNAs in a single‐ sequencing run.

**Table 1:** Assembly statistics from metagenomic reads

Number of contigs	3,811
Total size of contigs (nt)	2'853,727
Size of longest contig (nt)	37,904
Number of contigs > 1 kb	343
Number of contigs > 10 kb	37
Mean contig size (nt)	749
N50 contig size (nt)	1006
L50 contig count (nt)	337

### 3.2 Enzyme predictions

The gene and ORF prediction analyses on the entire metagenomic assembly identified a total of 105,773 putative proteins. In order to reduce redundancy in the database (two gene predictors yielding the same prediction), we clustered our results at 90% similarity along the entire protein prediction, generating 97,317 clusters. A peptide from each cluster (or seed) was chosen to represent the cluster and was used for further analyses. These seeds were on average 136 aa, with a standard deviation of 120 aa. Longest seed was 1,536 aa. Comparison of these seeds against PFAM database showed that only 2,202 had a PFAM hit. This represents only ~2% of the entire seeds used for analysis and it manifests the current limitations of functional annotation in metagenomes (Lobb et al., 2015), in which the great majority of predicted proteins have no homolog in databases. This result could also be partially caused by the parameters used in our gene prediction phase, in which we considered peptides of at least 30 aa long. In our analysis, almost all protein predictions in the metagenome are unique (singletons or doubletons), with only a few clusters having a significant number of members. This is an indication of the low sequencing depth and the high diversity of the soil metagenome. The fact that only a minor fraction of the predictions ended up having a hit in a database of domain assignment shows how little we know about potential new protein families in metagenomes, their potential novel functions, and the biases present in databases (Prakash & Taylor, 2012). Despite this fact, most of the PFAM hits corresponded to known protein families and only 6% of all the PFAM hits corresponded to domains of unknown function. All results were organized and filtered according to PFAM function. Sequences related to domains of lipases/esterases and proteases were selected for further analyses and selected as candidates for gene expression.

### 3.3 Functional profiles of predicted proteins

The 2,202 peptides with significant hits against PFAM represent 1,175 different protein families (domains), revealing an approximated ratio of 2:1 of predicted peptide:PFAM family. This shows that our library is far from functional saturation and indicates that this soil metagenome requires sequencing depths several orders of magnitude greater than the one used in this study.

**Figure 2:**
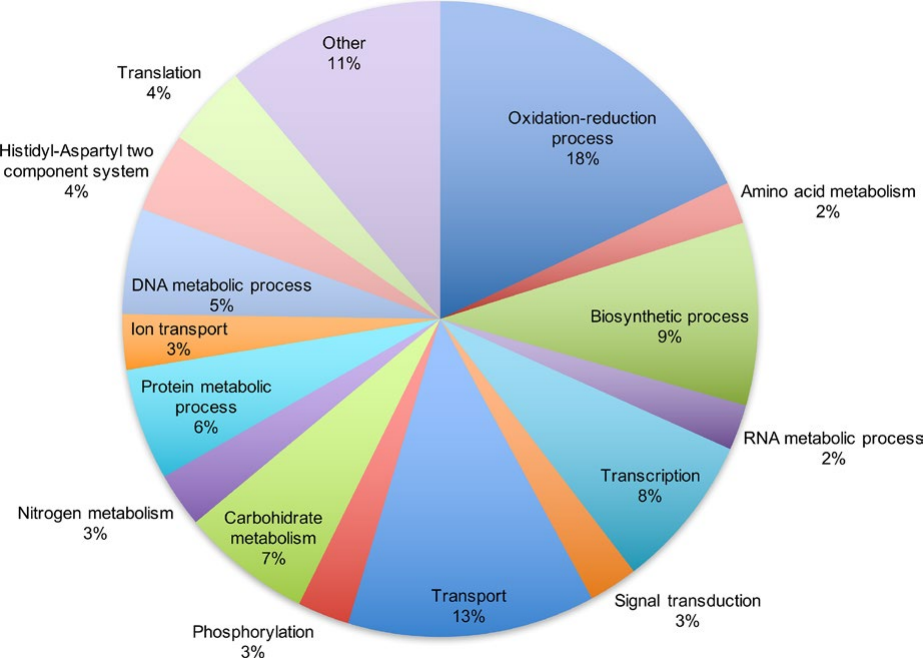
Gene Ontology functions of the annotated fraction of the metagenome. Proteins with associated PFAM domains were mapped to Gene Ontology terms (GOSlim). Most of the terms are associated with energy metabolism and transport in and out of the cell. Proteins can be binned into more than one category and therefore the total number of annotations is higher than the total number of proteins

To determine functional enrichment of the metagenome, we mapped proteins with PFAM hits against Gene Ontology terms (GoSlim). Results of these analyses are shown in Figure 2, where the most abundant molecular function term is related to oxidoreductase activity (18%), indicative of aerobic metabolism and consistent with the well‐aerated soils sampled in this study. Other abundant terms were related with the metabolism of carbohydrates (7%), protein metabolism (6%), nitrogen (3%), and transport of nutrients (13%), all related with energy metabolism. In the TAG‐assigned fosmids (Table S2), we identified that 14 out of 451 putative proteins (3.1%) included a protease domain and 12 (2.7%) included a lipase/esterase domain (Table 2), showing the relative scarcity of these enzymes with respect to proteins involved in the metabolism of energy.

**Table 2:** Metagenomic‐derived coding genes for putative lipases/esterases and proteases

Enzymes	Fosmid ID	Contig ID	Putative Gene	Size (nt)	Protein Size (aa)
Lipases/Esterases	F2	C14	Consensus_gene_329	1,116	371
	F2	C14	Consensus_gene_353	852	283
	F2	C14	Consensus_gene_354	288	95
	F5	C17	Consensus_gene_420^a^	2,115	704
	F6	C8	Consensus_gene_211	981	326
	F6	C8	Consensus_gene_212	651	216
	F8	C18	Consensus_gene_436	2,028	675
	F19	U17	U_42	1,086	361
	F25	C16	Consensus_gene_396	636	211
	F27	C3	Consensus_gene_87	792	263
	F28	U36	U_195	1,575	524
	F36	C25	Consensus_gene_553	600	199
Proteases	F5	C17	Consensus_gene_420	2,115	704
	F8	C18	Consensus_gene_436^b^	2,028	675
	F11	C20	Consensus_gene_472	435	144
	F11	C20	Consensus_gene_473	828	275
	F14	C15	Consensus_gene_359	1,098	365
	F21	U26	U_145	645	214
	F22	C5	Consensus_gene_122	1,278	425
	F22	C5	Consensus_gene_126	1,404	467
	F27	C3	Consensus_gene_62	1,707	568
	F27	C3	Consensus_gene_85	1,377	458
	F35	U21	U_70	849	282
	F36	C9	Consensus_gene_224	1,902	633
	F36	C9	Consensus_gene_232	1,146	381
	F38	C21	Consensus_gene_496^c^	1,101	366

a Gene encoding for protein denominated as LipM.

b Gene encoding for protein denominated as Prot1.

c Gene encoding for protein denominated as Prot2.

**Figure 3:**
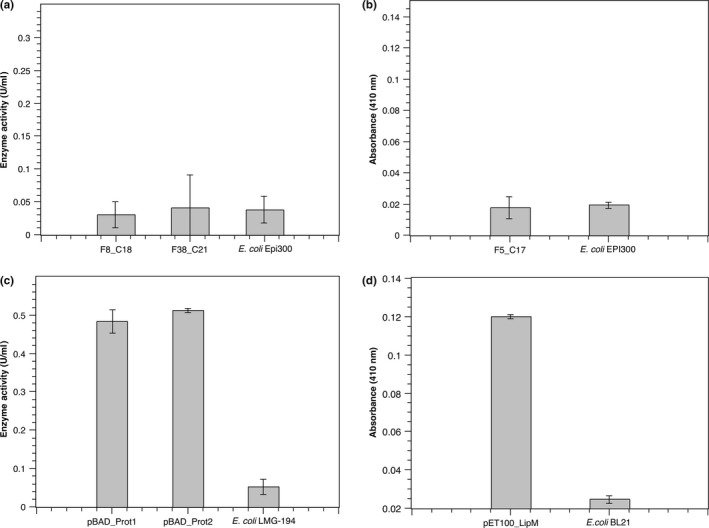
Bacterial enzymatic activity. (a) Proteolytic activity determination by the colorimetric method of Folin Ciocalteu reagent using casein as substrate of the reaction together with bacterial extracts from *E. coli* EPI300 metagenomic clones F8_C18 (harboring Prot1 CDS) or F38_C21 (harboring Prot2 CDS). (b) Lipolytic activity detection by *p*‐Nitrophenyl butyrate degradation of the bacterial extract derived from *E. coli* EPI300 metagenomic clone F5_C17 (harboring LipM CDS). In (a) and (b) *E. coli* EPI300 was used as a negative control of the enzymatic activities. (c) Proteolytic activity determination of bacterial extracts derived from *E. coli* LMG‐194 clones harboring either pBAD_Prot1 or pBAD_Prot2. (D) Lipolytic activity detection of the bacterial extract derived from *E. coli* BL21 harboring pET100_LipM plasmid. In (c) and (d), the respective nontransformed *E. coli* strains were used as negative controls of enzymatic activity. Error values represent standard deviations from three replicates in each case. *Indicates a significant difference in the proteolytic activity from clones pBAD_Prot1 and pBAD_Prot2 (*p*‐value < .05) compared with negative control. **Indicates a significant difference in the lipolytic activity of the bacterial extract derived from clone pET100_LipM (*p*‐value < .05) compared with negative control

Based on this information and to assess the capacity of the predicted putative foreign genes to express their associated phenotypes, the analyses were restricted to identify only lipases/esterases and proteases in certain metagenomic clones (*E. coli* EPI300 harboring fosmids F5_C17, F8_C18, and F38_C21 from Table 2). Functional analyses in agar plates supplemented with their respective substrates did not show the expected halo formations, as a result of the substrate degradation, from any of the originally selected metagenomic clones, each one harboring either Contig C17, C18, or C21 (data not shown). It has been reported that one of the main disadvantages of these direct detection/screening methods is their low resolution and sensitivity, resulting in no detection of metagenomic clones that exhibit low expression levels of the desired enzymatic activities (Uchiyama & Miyazaki, 2009). These traditional strategies are also highly restricted to the detection of enzymes and compounds secreted to the surrounding culture media by the bacterial host. In consequence, we used a more sensitive approach for *in vitro* detection of both phenotypes in the metagenomic clones. Specifically, the lipolytic activity was assessed based on the degradation of *p*‐Nitrophenyl butyrate, while proteolytic activity was measured after quantifying the release of tyrosine from casein as substrate. Despite these approaches, none of the selected metagenomic clones containing a protease or a lipase/esterase putative sequences (*E. coli* EPI300_ F5_C17: LipM; *E. coli* EPI300_ F8_C18: Prot1; and *E. coli* EPI300_ F38_C21: Prot2) exhibited higher enzymatic levels than those registered by the respective negative controls used in the experiments (Figure 3a and b).

These results show the limitations of heterologous gene expression, in this case, a bacterial host unable to express genes predicted to encode for proteases Prot1, Prot2, and the lipase/esterase LipM, in context of metagenomic DNA. These observations are consistent with the reported problems of *E. coli* to recognize and express the majority of genes present in foreign DNA inserts (Ekkers et al., 2012). Subsequent analysis of the up‐stream region of the three enzyme‐coding sequences suggested that the lack of expression could be due to the failure of the *E. coli* transcriptional machinery to recognize the foreign DNA promoter regions. The selectivity of the bacterial host to recognize promoter regions has been well documented (Warren et al., 2008), and is one of the main reasons resulting in scarce identification of enzymes and metabolites in metagenomic functional screening assays (Gaida et al., 2015).

### 3.4 Recovery of enzymatic activities and characterization

The open reading frames (ORFs) encoding for the selected enzymes (Prot1, Prot2 and LipM) were amplified from its corresponding metagenomic clone and subcloned in *E. coli* expression vectors (pET100/D‐TOPO or pBAD/gIII). The assessment of lipolytic and proteolytic activities from the subclones revealed the expected enzymatic functions (Figure 3c and d). These assays validated the *in silico* characterization of putative proteins in metagenomic DNA and suggested that the previous nondetection of activities in the original metagenomic clones was due to heterologous expression impairments of the genes located inside the foreign DNA fragments. In this case, the selected enzyme‐coding sequences were intact during the subcloning steps in the expression vectors, which in turn might indicate that the bottleneck for the individual functional gene expression in the metagenomic clones took place probably at the transcriptional level.

The nucleotide BLAST performed for each of the protein coding sequences for Prot1, Prot2, and LipM showed no match in GenBank, using the nonredundant database for all the organisms. Protein homology by BLAST using the related amino acid sequences of the three proteins showed different results. Sequence of Prot1 showed 70% identity with an aminopeptidase of *Chthoniobacter flavus*, a bacterium belonging to the phylum Verrucomicrobia (Kant et al., 2011). Analyses of Prot1 in PFAM and MEROPS databases showed homology with M29 protease superfamily. Prot2 showed 65% identity with S9 peptidase family of *Fischerella sp*. (Prosperi et al., 1992). This family of proteases mainly contain serine proteases as well as propyl endopeptidases, enzymes specialized in the cleavage of proteins toward their C‐terminus, specifically in proline residues (Fülöp et al., 1998). On the other hand, LipM protein sequence exhibited 48% identity with the Alpha/beta hydrolase AS‐Trib12 belonging to an uncultured bacterium. Although these homology analyses were carried out with proteins that were identified from already reported domains, it is surprising to observe identity values even much lower than those observed for novel enzymes recovered in functional assays (67–92% identity with >90% query cover) (Biver, Portetelle, & Vandenbol, 2013; Devi et al., 2016). This result highlights even more the impact of the current approach to identify hidden novel enzymes from metagenomic samples.

**Figure 4:**
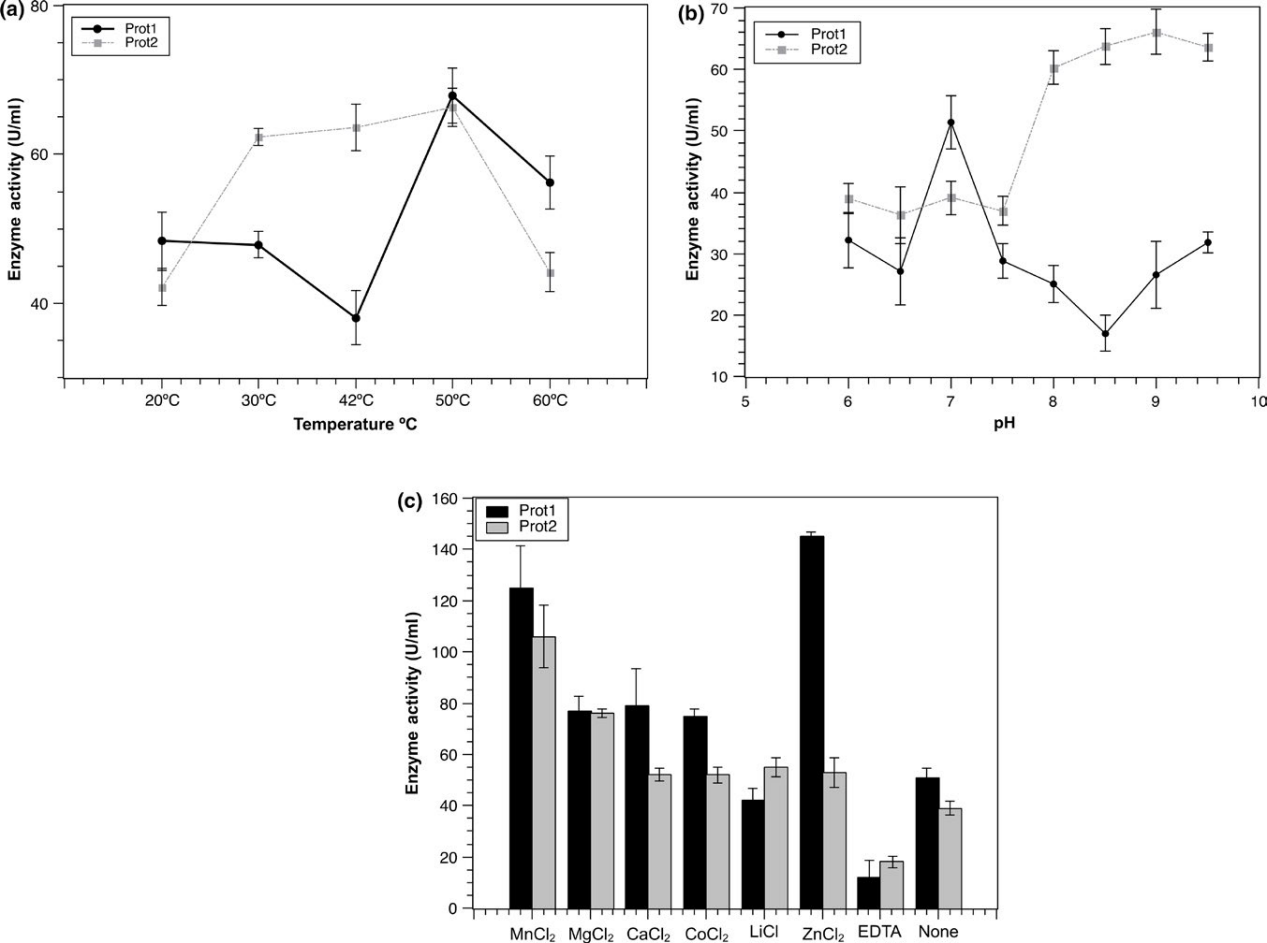
Partial protease characterization. (a) Effect of temperature on protease activities of Prot1 and Prot2. (b) Effect of pH on protease activities of Prot1 and Prot2. (c) Effect of metal ions and inhibitor (EDTA) on the enzymatic activities of Prot1 and Prot2

The further enzyme characterizations were only performed with the two proteases. The enzymatic activities of Prot1 and Prot2 reached highest activity at 50°C, which matches with activity reports for metalloproteases and serine proteases, respectively, obtained from metagenomic libraries (Lee et al., 2007; Rao et al., 1998) (Figure 4a). Interestingly, at the highest temperature assessed (60°C), both proteases still exhibited significant activity values, which could be relevant for industrial applications, like detergent production and laundry processes (Devi et al., 2016). Prot1 enzyme had the highest performance in neutral pH values (7.0 to 8.0), while Prot2 showed greater activity in alkaline pH values (8.0 and 9.5) (Figure 4b). Additionally, enzymatic analyses including different metal ions were also assessed for these two enzymes (Figure 4c). Prot1 exhibited an increased activity with Mn^2+^ and Ca^2+^, while Prot2 exhibited an increased activity with Mn^2+^, Ca^2+^ and Zn^2+^. In addition, the incubation of both enzymes with EDTA reduce significantly their activities, which suggests once more the closer link between these enzymes with metalloproteases and serine proteases (Pushpam, Rajesh, & Gunasekaran, 2011; Waschkowitz, Rockstroh, & Daniel, 2009). Several authors report that the use of metal ions such as Co^2+^, Fe^3+^, Zn^2+^, Mn^2+^, Co^2 +^, and Ca^2+^ could protect these types of proteases from thermal denaturation and may play an important role in the maintenance of their conformation at high temperatures (Kasana, Salwan, & Yadav, 2011).

In a recent report, Ferrer and coworkers have estimated the success of enzyme bioprospecting through metagenomics (Ferrer et al., 2015). They showed that the success in isolating proteases has a ratio of 1:9,833 screened clones, while for lipases/esterases the ratio is 1:17,320 screened clones. This efficiency in recovering metagenomic enzymes contrasts with our strategy in which we identified one lipase/esterase and two proteases from only 40‐screened clones. On the other hand, direct sequencing of a complete metagenome, although very attractive, is a challenging task. There is still a lack of reliable bioinformatics pipelines for analysis of next‐generation sequencing data, in order to (1) correctly assemble the huge diversity of genome fragments from complex DNA samples and to (2) avoid the potential formation of chimeric contigs (Ghosh, Mehra, & Mande, 2015; Nyyssönen et al., 2013).

Different functional metagenomic studies have led to the development of diverse tools to counteract the difficulties associated with the low or null transcription of foreign genes in a metagenomic context. Some of these strategies include the development of plasmids harboring flanking lac‐promoters (Lämmle et al., 2007) or fosmids and cosmids harboring viral‐related promoters (Lussier et al., 2011; Terrón‐González et al., 2013), bacterial hosts coexpressing heterologous sigma factors (Gaida et al., 2015), and the random insertion of promoters in metagenomic DNA by the use of transposons (Leggewie et al., 2006). Although these approaches have partially improved the enzymatic detection in metagenomes, we consider that the pipeline presented here demonstrates that pooled fosmid sequencing followed by *in silico* prediction analyses of putative genes can be a powerful and cost‐effective way to efficiently recover functional enzymes, making it suitable as part of the metagenomic toolbox for identification and characterization of hidden activities in metagenomic libraries.

## CONFLICT OF INTEREST

None declared.
